# Development and validation of a nursing professionalism evaluation model in a career ladder system

**DOI:** 10.1371/journal.pone.0186310

**Published:** 2017-10-12

**Authors:** Yeon Hee Kim, Young Sun Jung, Ja Min, Eun Young Song, Jung Hui Ok, Changwon Lim, Kyunghee Kim, Ji-Su Kim

**Affiliations:** 1 Department of Nursing, Asan Medical Center, Seoul, Republic of Korea; 2 Department of Applied Statistics, Chung-Ang University, Seoul, Republic of Korea; 3 Red Cross College of Nursing, Chung-Ang University, Seoul, Republic of Korea; Waseda University, JAPAN

## Abstract

**Background:**

The clinical ladder system categorizes the degree of nursing professionalism and rewards and is an important human resource tool for managing nursing.

**Aim:**

We developed a model to evaluate nursing professionalism, which determines the clinical ladder system levels, and verified its validity.

**Methods:**

Data were collected using a clinical competence tool developed in this study, and existing methods such as the nursing professionalism evaluation tool, peer reviews, and face-to-face interviews to evaluate promotions and verify the presented content in a medical institution. Reliability and convergent and discriminant validity of the clinical competence evaluation tool were verified using SmartPLS software. The validity of the model for evaluating overall nursing professionalism was also analyzed.

**Results:**

Clinical competence was determined by five dimensions of nursing practice: scientific, technical, ethical, aesthetic, and existential. The structural model explained 66% of the variance. Clinical competence scales, peer reviews, and face-to-face interviews directly determined nursing professionalism levels.

**Conclusions:**

The evaluation system can be used for evaluating nurses’ professionalism in actual medical institutions from a nursing practice perspective.

**Implications for nursing management:**

A conceptual framework for establishing a human resources management system for nurses and a tool for evaluating nursing professionalism at medical institutions is provided.

## Introduction

Recently, in response to the opening of medical markets and institutional accreditation assessments, medical departments have found it necessary to seek innovative strategies to enhance healthcare services and strengthen international competitive advantages. While investing in various physical resources and facilities is an important practice, securing and managing excellent human resources has emerged as the most critical factor for achieving such goals. Nurses constitute the largest portion of the professional staff at medical institutions and continuous effort is required to promote competency in the nursing profession to meet the elevated expectations related to cost-effectiveness, high-quality nursing services, and the societal demands placed on nurses [1]. Professionalism is an important feature of the work, which is determined by three attributes: cognitive, attitudinal, and psychomotor [2]. According to Coulon et al. (1996), nursing professionalism incorporates the “way in which nurses enacted their caring role with patients” [3]. Therefore, to strengthen competency in the nursing profession, and to promote experienced clinical nurses to provide quality care and to accomplish the results expected by nursing, sufficient skills and qualifications must first be applied to core nursing practices based on a theoretical understanding across nursing. For this reason, core levels of nursing competency that must be achieved at certain career points is suggested, beginning with new nurses. Furthermore, educational conditions and environments for achieving such goals should be provided, along with an evaluation of methods required for nurses’ professional competency and experience.

### Background

The career ladder system (CLS) is a systematic and strategic human resource management method that improves the quality of patient nursing by advancing the reward and recognition of experience and competency achieved by nurses in clinical practice [4]. CLS is very effective and plays a vital role for both individuals and organizations, allowing for evaluation of and compensation for nursing professionalism. Such career ladder programs simultaneously satisfy the demand for individual growth and maximize human resources development in an organization. Therefore, these programs have a positive effect on harmonizing individuals and organizational demands [5]. Specific CLS effects include job satisfaction [6], organizational commitment [7], professional growth [8], increased quality of nursing [8], reduced turnover [6, 7], cost-effectiveness [6], and improved leadership [9]. Accordingly, medical institutions globally apply CLS as part of motivation programs for developing nurse professionalism [10]. Such programs have been developed over extended periods to reflect the career demands of nurses; continuous evaluation and revision is achieved through effective communication during implementation. However, there is a general lack of understanding regarding CLS in the South Korean medical field; only a very limited number of medical facilities are implementing the system due to inadequate administrative and financial support [11].

The study was conducted at hospital A; a CLS was developed with compensation ranges divided into four levels by nurses’ clinical experience, education, competency, and professionalism, thereby supporting their own efforts for development. Furthermore, continuous evaluations and revisions were performed in the CLS to establish a comprehensive human resource management system for nursing staff in a clinical setting. The result of the current career ladder programs evaluation at hospital A showed no direct dissatisfaction toward the program by nurses or comments for improvements; however, nurses expected various evaluation methods, including a peer review system. The need for an objective method to evaluate professionalism was suggested [11].

Professional nursing is the practice of conducting appropriate nursing tasks in response to patients by utilizing technology, science, and theories in a clinical setting [12]; evaluations of practice are required to objectively assess professionalism. Therefore, assessment of various nursing competencies in scientific, technical, ethical, aesthetic, and existential dimensions [13] of clinical practice remains necessary to evaluate professionalism in a creative, innovative, and original way. If such problems are solved, then a standardized quantitative and qualitative evaluation of nursing practice becomes possible, and can be used as an indicator of an objective CLS that assesses nursing professionalism. Additionally, this process can provide basic data in the development of nursing career ladder programs and application processes to inform future clinical settings. For this reason, this study developed a CLS evaluation model of nursing professionalism to objectively evaluate nurses’ competency during service, and verified the validity of the model.

**Fig 1:**
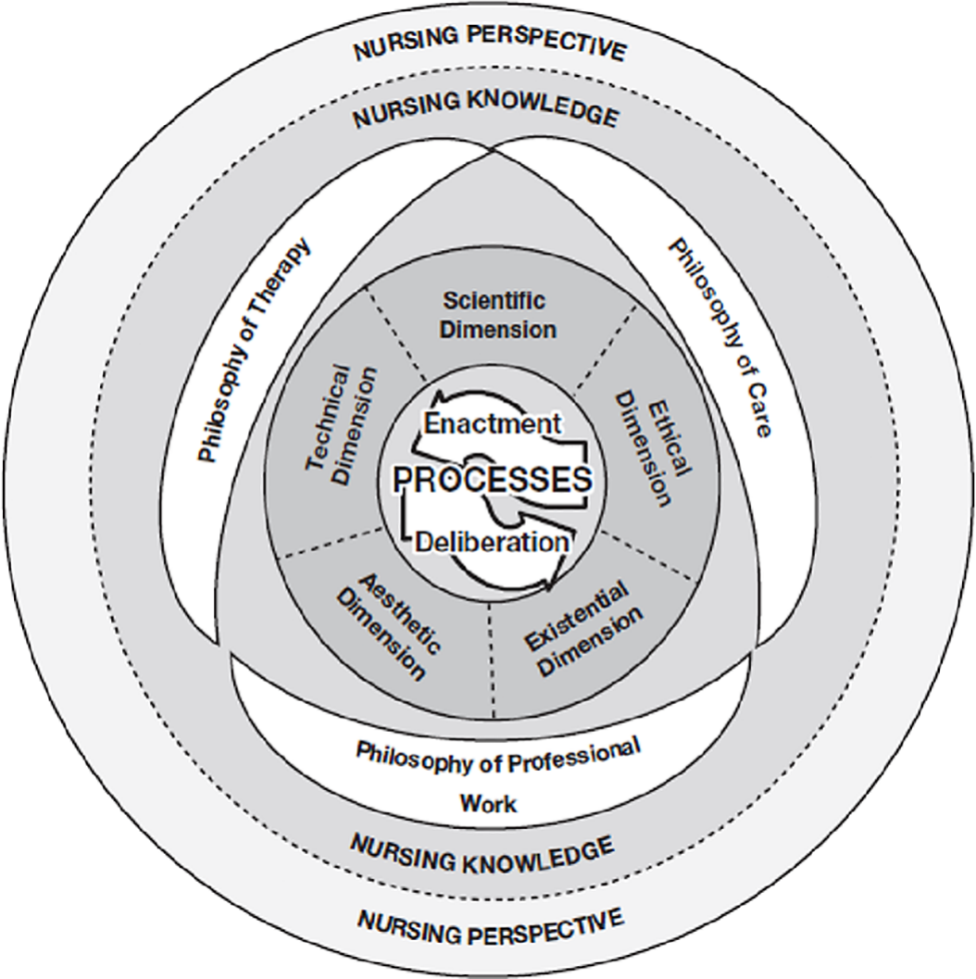
Model of nursing practice.

We constructed a model to assess nursing professionalism using a clinical competence scale to evaluate promotion case reports; scientific, technical, ethical, aesthetic, and existential dimensions were categorized using Kim’s (2010, 2015) nursing practice model [13, 14] **(Fig 1)**. Then, a nursing professionalism evaluation model that employed multi-rater feedback of clinical competence, peer reviews, and face-to-face interviews to verify its validity was established. Specific study objectives were as follows: (1) develop preliminary questions to measure clinical competence; (2) verify the validity of the preliminary questions; (3) confirm reliability among the measurement variables in the developed questions; (4) verify the construct, convergent, and discriminant validity of the developed questions; and (5) confirm the validity of the nursing professionalism evaluation model using partial least squares structural equation modelling (PLS-SEM).

## Methods

### Ethics statement

This study received ethical approval (No. 2013–1057) from the institutional review board of Asan Medical Center. After explaining the study’s purpose and content, written consent was collected from all participants.

### Study design

**Fig 2:**
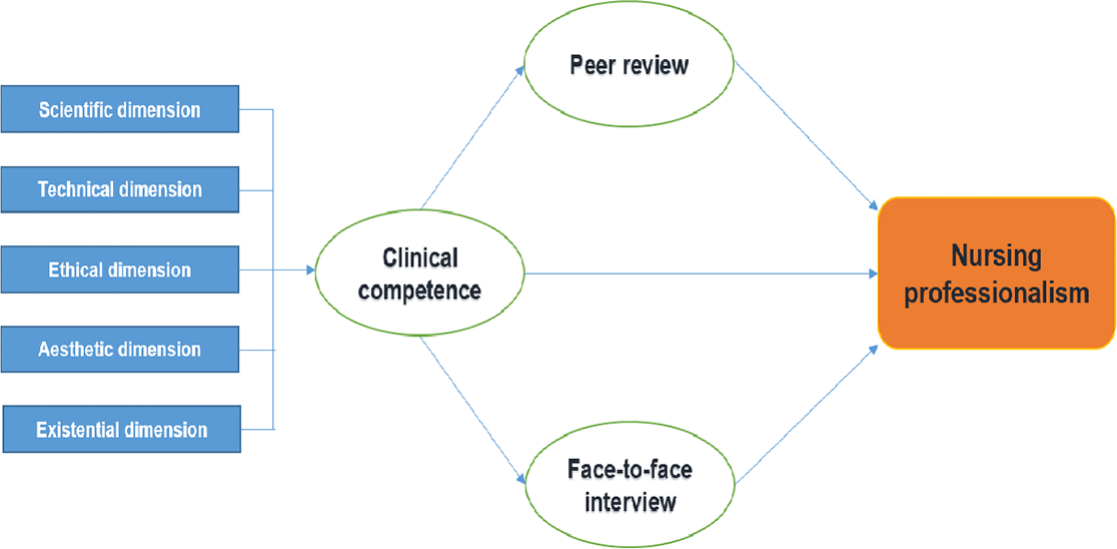
Conceptual framework.

The conceptual framework of the nursing professionalism model verified in this study is shown in Fig 2. Nursing professionalism, which is a dependent latent variable, is evaluated through independent latent variables, such as clinical competence, peer reviews, and face-to-face interviews for each nursing dimension. Formative measurements were used in this study to form the clinical competence measurement model. The only criteria for determining whether to use reflective or formative measurement to develop the measurement model was the auxiliary theory [15]. Formative measurement contains two types of basic assumptions: (1) the measurement variable becomes the cause of the construct [16] and (2) measurement error cannot completely explain the construct [17]. For example, if productivity, profitability, and market share are defined as an organization’s performance, formative measurement should be selected since they reflect distinct aspects [18]. As the five nursing dimensions do not present similar attributes, but rather reflect different clinical competence aspects, formative measurement was used as the model in this study. Therefore, the model for evaluating professionalism in nursing proposed in this study is a hierarchical latent variable model that uses a reflective-formative type method; the repeated indicator approach was applied to estimate clinical competence, such as the second order [19]. The repeated indicators approach re-assigned the measurement indicators from the first order to the second order to conduct simple estimates for the second order [18].

### Participants

Participant data were randomly extracted from 200 cases (all women, mean age = 31.27 ± 4.12 years). Eighteen graduated from a three-year college (8.8%), 145 graduated from a four-year college (71.1%), and 37 were enrolled in a graduate school program or above (18.1%). The range of clinical experience at the corresponding hospital was 40 to 267 months, with an average duration of 102.85 ± 49.10 months.

Half were passing case reports and half did not pass the CLS level II to III promotion review between 2010 and the first half of 2014 at nursing departments in a tertiary general hospital located in Seoul, South Korea. The number of participants was higher than the recommended number of 154, calculated by a medium effect size of 0.15 and power of 0.90, which is required for multiple regression analyses using G-power 3.0 analysis software [20]. Therefore, the sample size used in this study was sufficient for statistical power. The participating medical institution was a large hospital in Seoul comprising approximately 2,700 beds, 1,500 doctors, and 2,900 nurses; there were 1.57 patients per nurse. Participants’ evaluation scores for nursing professionalism, face-to-face interviews, and peer reviews were used for this study. Additionally, the clinical competence tool developed to evaluate promotion case reports was used to evaluate clinical competence scores of the 200 cases.

### Measures

In this study, a clinical competence scale was developed for constructing and verifying the nursing professionalism evaluation model. Two hundred extracted case reports were re-evaluated using the developed tool. Nursing professionalism (item-level content validity index [I-CVI] 0.94, scale-level content validity index average [S-CVI/Ave] 0.94), face-to-face interview (I-CVI 0.94, S-CVI/Ave 0.94), and peer review (I-CVI 1.00, S-CVI/Ave 1.00) scores were measured using a valid evaluation tool for CLS promotion reviews by the corresponding institution (Table 1).

**Table 1:** Measurement tool items.

Variable	Dimension	Item	
Clinical competence	Scientific	Is subjective and objective data of the client accurately collected?	X11
		Is the client’s health problem deduced in a logical manner?	X12
		Is it a reasonable care plan for the client?	X13
		Is care based on scientific evidence being provided to the client?	X14
		Does the client show reasonable evaluation of the expected results?	X15
	Technical	Are the resources necessary for treating the client used in an effective manner?	X21
		Is nursing being performed in an experienced manner?	X22
		Is the proper action being completed according to the priorities in response to the changing state of the client?	X23
		Is the necessary personalized training being provided to the client/guardian?	X24
	Ethical	Is the correct decision being made so no harm results for the client?	X31
		When performing care for the client, is moral autonomy being exercised as a nurse?	X32
		Is the role of the client’s advocate being practiced?	X33
		Is mutual respect and a trusting relationship being formed with the nursing client?	X34
	Aesthetic	Am I practicing empathy and reacting sensitively to the client?	X41
		Am I providing care that harmonizes with the specific clinical circumstances and the individuality of the client, and in doing so, does the client feel beautiful?	X42
		Am I practicing leadership that can lead to positive change?	X43
	Existential	Do I have a holistic understanding of the client?	X51
		Am I practicing self-reflection in caring for the client?	X52
		Am I practicing self-development as a professional nurse?	X53
Face-to-face interview		Motive for the application and aspirations	X61
		Contribution to the department	X62
		Future plans (growth potential)	X63
		Nursing philosophy (conviction)	X64
Peer review	Patient care	Utilize nursing knowledge, make clinical decisions, and analyze critical situations.	X71
		Appropriately use every resource at your disposal to establish care plans for clients in the entire team.	X72
		Operate machinery and equipment related to work in the correct manner, and help colleagues.	X73
	Human resources development	Prepare yourself to teach clinical training in the nursing unit, and support the growth of colleagues and juniors as a preceptor or a mentor.	X74
	Leadership	Provide opinions about effective operation of the nursing unit, actively participate in decisions, and take responsibility.	X75

First, Kim’s (2010, 2015) nursing practice model [13, 14] was applied to develop a clinical competence scale to measure clinical competence from scientific, technical, ethical, aesthetic, and existential dimensions of nursing professionalism. Focus group interviews were conducted with 20 CLS level III nurses recommended by the department heads of the corresponding institution. They voluntarily signed written consent to participate in the study to construct preliminary questions through interview analysis along with the existing literature review. To assess the content validity of the preliminary questions, a group of nursing professionals was created consisting of 21 CLS evaluation committee members. An I-CVI score of 0.96 and S-CVI/Ave score of 0.96 verified the content validity of the questions [19]. Nineteen questions underwent final inspection from a Korean language and literature professor to verify sentence validity. The 19 verified questions with content validity belonged to five dimensions—scientific (five items), technical (four items), ethical (four items), aesthetic (three items), and existential (three items)—with each clinical competence item evaluated on a five-point scale and a full range of 19–95 points. Case reports of the 200 promotional cases were evaluated through the clinical competence tool development. Pairs of people confirmed each other’s reliability, with 20 evaluators reviewing 10 case reports.

For the face-to-face interviews, evaluations were performed while the committee member conducted an individual interview with the nurses applying for the CLS level III promotion. The four verified questions on this topic were (1) application motive and applicant aspirations, (2) department contribution, (3) future plans and development potential, and (4) nursing philosophy. Each item was evaluated on a 5-point scale with a range of 4–20 points.

Peer review was only performed when the average score of the case report applying for the CLS level III promotion was above 70 points. For the content validity-verified peer review score, the average score of six peers for each applicant was used. The review comprised five questions: three related to patient care, one on talent development, and one on leadership. The questions were evaluated on a four-point scale with a total range of 4–20 points.

### Statistical analysis

Second-generation statistical techniques were used to model simultaneous relationships among multiple constructs [21]. To verify the reflective-formative type model proposed in this study, the hierarchical latent variables model using PLS-SEM [22] was used. PLS-SEM is primarily intended to examine complex relationships between latent constructs in structural models [23]. It can be used in both exploratory and confirmatory studies [24]. SmartPLS (version 2.0 beta) was used for the data analysis [24].

PLS-SEM was performed on the research model (Fig 2). First, latent constructs were selected following the theoretical constructs within the model (scientific, technical, ethical, aesthetic, and existential dimensions; clinical competence; face-to-face interview; peer review; and nursing professionalism). Then, indicators for each latent variable were included. Those that reached a .05 significance level using bootstrapping were retained. Simultaneously, relationships between latent variables were examined until the best predictive model was obtained. Confidence intervals of the PLS-SEM coefficients were obtained by cross-validation.

Outliers and missing values of all variables were confirmed prior to data analysis. As peer review was not conducted for applicants for CLS level III promotion with a score below 70 from the case report evaluation tool, missing peer review values became systematic missing data. Therefore, standard imputation methods, such as the regression imputation, stochastic regression imputation, and Bayesian imputation commonly used for random missing values, could not be used. Additionally, the use of a removal method eliminated most CLS level III promotion applicants for whom correct analysis could not be conducted.

For this reason, the following method was used to generate scores for each item as a substitute. (1) As promotion applicants with an evaluation score below 70 did not receive a peer review score, it was assumed that the peer review score would be lower than 80 if they underwent peer review (applicants with a peer review score above 80 points were promoted). (2) Under that assumption, the average peer review score by item was calculated from the normal distribution values for participants who did not undergo peer review. (3) Scores corresponding to each item were not replaced with an identical mean value for all participants who did not have a peer review score; however, the distribution of question scores for participants with peer review scores were calculated. It was assumed that the calculated average for each item followed a normal distribution. The distribution of the scores was generated randomly.

### Ethical considerations

This study received ethical approval (no. 2013–1057) from the institutional review board of A Medical Center. After explaining the study’s purpose and content, written consent was collected from all participants. The research team stored the originals; copies were provided to the researchers and study participants. Personal information from participants was stored in a locked filing cabinet, to be disposed three years after the end of the study to maintain confidentiality.

## Results

### Measurement model

The reliability and validity of the measures used for each construct were tested in the measurement model (Table 2). Internal consistency reliabilities were all at least 0.85, exceeding the minimal reliability criteria of 0.70 [25]. Additionally, average variance extracted (AVE) exceeded .50 for each measure, indicating that at least 50% of the variance of each latent variable was explained by its contributors [23]. The elements in the matrix diagonal of Table 2, representing the square roots of the AVEs, are greater in all cases than the off-diagonal elements in their corresponding rows and columns, except for the aesthetic, technical, and ethical dimensions, supporting discriminant validity at the latent variable level. In other words, in the latent variable intercorrelations, correlations between aesthetic and technical dimensions and between aesthetic and ethical dimensions were slightly higher than the correlation between the technical and ethical dimensions in the corresponding row.

**Table 2:** Reliability, convergent and discriminant validity, and correlations among latent constructs in the measurement model.

Latent Construct	Items	ICR^a^	SD	TD	ED	AD	ExD	FFI	PR	NP	AVE	Cronbach’s α
Scientific dimension (SD)	5	0.93	**0.85**^b^								0.72	0.90
Technical dimension (TD)	4	0.87	0.78	**0.79**^b^							0.62	0.79
Ethical dimension (ED)	4	0.88	0.77	0.78	**0.80**^b^						0.64	0.81
Aesthetic dimension (AD)	3	0.89	0.74	0.80	0.82	**0.85**^b^					0.73	0.81
Existential dimension (ExD)	3	0.81	0.67	0.61	0.65	0.73	**0.77**^b^				0.59	0.66
Face-to-face interview (FFI)	4	0.85	0.18	0.22	0.21	0.24	0.24	**0.77**^b^			0.59	0.77
Peer review (PR)	5	0.99	0.15	0.24	0.24	0.22	0.19	0.28	**0.98**^b^		**0.96**	0.99
Nursing professionalism (NP)	1	1.00	0.30	0.39	0.40	0.40	0.33	0.41	0.73	**1.00**^b^	**1.00**	1.00

^a^ICR = internal consistency reliability

^b^AVE test values

Factor and cross-loadings of all indicator items to their respective latent constructs were extracted to test convergent validity. While there was no set range or minimum, the narrower the range and higher the lowest loading, the more convergent validity could be assumed [23]. The results indicated that all items loaded on their respective constructs from a lower bound of .66 to an upper bound of .98, and more highly than on any other constructs (Table 3).

**Table 3:** Factor structure matrix of loadings and cross-loadings in the measurement model.

Factor	SD	TD	ED	AD	ExD	FFI	PR	NP
Scientific dimension (SD)								
X11	**0.85**^**a**^	0.67	0.65	0.63	0.57	0.20	0.12	0.24
X12	**0.88**^**a**^	0.68	0.66	0.64	0.60	0.12	0.17	0.27
X13	**0.87**^**a**^	0.72	0.69	0.69	0.59	0.17	0.12	0.27
X14	**0.86**^**a**^	0.67	0.66	0.61	0.54	0.12	0.12	0.26
X15	**0.77**^**a**^	0.55	0.62	0.57	0.56	0.18	0.13	0.22
Technical dimension (TD)								
X21	0.59	**0.78**^**a**^	0.58	0.63	0.37	0.21	0.17	0.26
X22	0.63	**0.83**^**a**^	0.65	0.65	0.50	0.14	0.17	0.27
X23	0.69	**0.82**^**a**^	0.65	0.61	0.49	0.20	0.24	0.35
X24	0.53	**0.71**^**a**^	0.56	0.63	0.55	0.14	0.19	0.34
Ethical dimension (ED)								
X31	0.61	0.64	**0.77**^**a**^	0.62	0.47	0.15	0.15	0.30
X32	0.63	0.60	**0.82**^**a**^	0.68	0.57	0.24	0.23	0.41
X33	0.68	0.68	**0.85**^**a**^	0.70	0.58	0.16	0.22	0.31
X34	0.54	0.57	**0.75**^**a**^	0.61	0.45	0.11	0.18	0.24
Aesthetic dimension (AD)								
X41	0.62	0.66	0.66	**0.83**^**a**^	0.62	0.20	0.12	0.27
X42	0.70	0.73	0.75	**0.89**^**a**^	0.67	0.21	0.20	0.38
X43	0.58	0.65	0.68	**0.84**^**a**^	0.56	0.20	0.24	0.37
Existential dimension (ExD)								
X51	0.61	0.64	0.64	0.66	**0.81**^**a**^	0.12	0.15	0.27
X52	0.49	0.39	0.42	0.54	**0.82**^**a**^	0.23	0.15	0.25
X53	0.41	0.31	0.38	0.45	**0.66**^**a**^	0.23	0.14	0.23
Face-to-face interview (FFI)								
X61	0.12	0.22	0.18	0.22	0.20	**0.73**^**a**^	0.24	0.28
X62	0.15	0.15	0.17	0.19	0.16	**0.81**^**a**^	0.21	0.36
X63	0.14	0.13	0.13	0.14	0.17	**0.77**^**a**^	0.21	0.33
X64	0.16	0.18	0.16	0.18	0.21	**0.77**^**a**^	0.21	0.27
Peer review (PR)								
X71	0.16	0.24	0.25	0.21	0.17	0.29	**0.98**^**a**^	0.74
X72	0.16	0.25	0.25	0.22	0.20	0.29	**0.98**^**a**^	0.72
X73	0.15	0.25	0.24	0.23	0.21	0.27	**0.98**^**a**^	0.73
X74	0.11	0.20	0.22	0.19	0.17	0.26	**0.98**^**a**^	0.69
X75	0.16	0.25	0.24	0.22	0.20	0.27	**0.98**^**a**^	0.71
Nursing professionalism (NP)								
Existing evaluation tool score	0.30	0.39	0.40	0.40	0.33	0.41	0.73	**1.00**^**a**^

^a^Loadings greater than .6 compared to loadings in other latent constructs.

### Structural model

**Fig 3:**
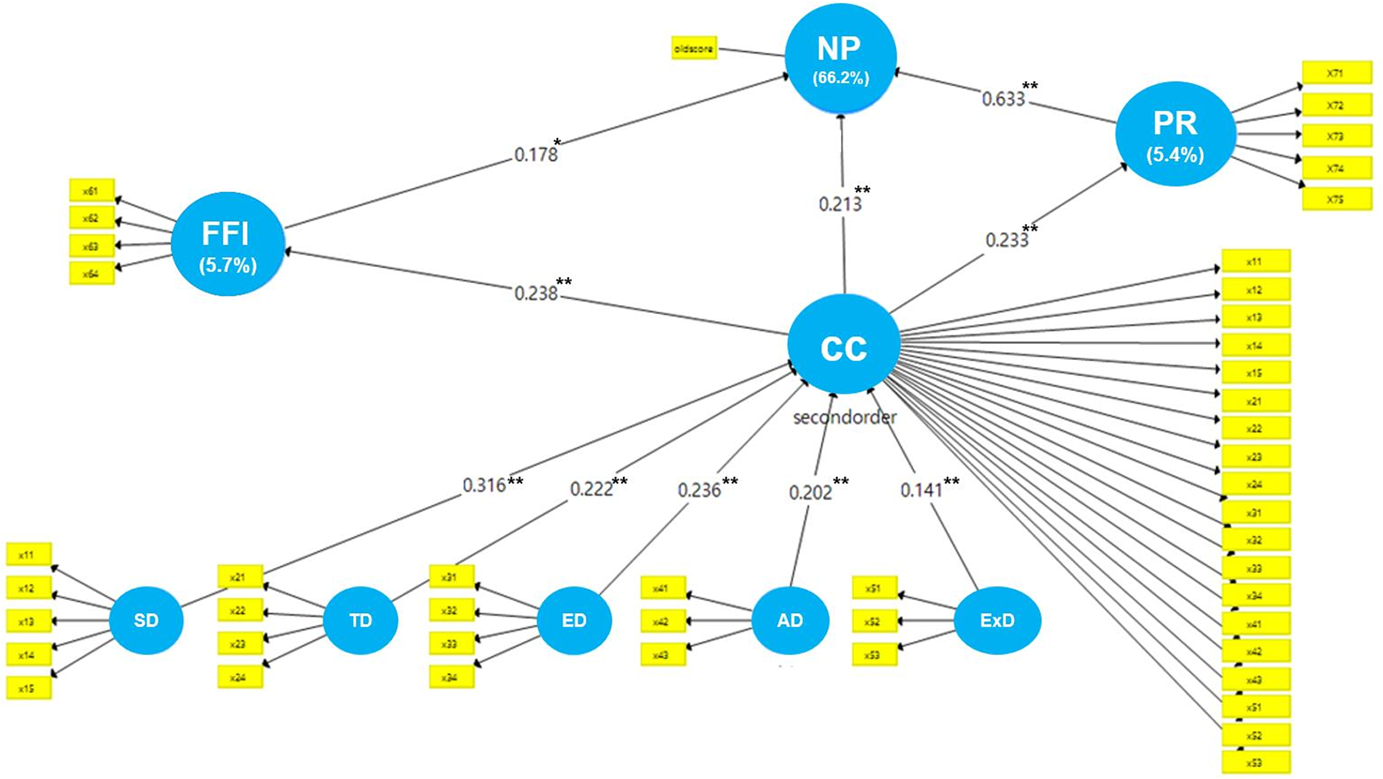
Structural model. SD = scientific dimension; TD = technical dimension; ED = ethical dimension; AD = aesthetic dimension; ExD = existential dimension; CC = clinical competence; FFI = face-to face interview; PR = peer review; NP = nursing professionalism. Explained variance (R2) is shown in parentheses. **p < .01; ***p < .001.

The structural model was assessed by examining path coefficients and their significance levels (Fig 3). The nursing professionalism level was determined directly through components of the clinical competence dimensions, face-to-face interview scores, and peer reviews. All β path coefficients were positive in the expected direction and statistically significant at p < .01 (Fig 3), indicating that they accurately estimated the relationship between constructs. First, the β path coefficients between second-order clinical competence measured with formative measurement and each dimension were as follows: scientific (β = 0.316, *p* < .001), technical (β = 0.222, *p* < .001), ethical (β = 0.236, *p* < .001), aesthetic (β = 0.202, *p* < .001), and existential (β = 0.141, *p* < .001). Furthermore, the β path coefficient between clinical competence and the face-to-face interview was 0.238 (*p* < .001), and the β path coefficient between clinical competence and peer review was 0.233 (*p* < .001), thus confirming the validity of the clinical competence scale to measure nursing service competence within each dimension. Moreover, the validity of the three evaluation procedures—clinical competence (β = 0.213, *p* < .001), face-to-face interview (β = 0.178, *p* = 001), and peer review (β = 0.633, *p* < .001)—were also confirmed. The predictive power of the model was tested through the estimation of R^2^ values. The explained variance of nursing professionalism by the three constructs was .622, indicating high explained variance of all constructs and supporting the model’s predictive value.

The impact of each latent variable on the corresponding dependent latent variable was examined through effect size (change in R^2^). According to the results in Table 4, the effect size of peer review on nursing professionalism was strong, while the effect sizes of face-to-face interviews and clinical competence scores on nursing professionalism were low to moderate [26]. Q2 indices exceeded the proposed threshold of Q2 > 0 [25] for clinical competence (.52), face-to-face interview (.03), peer review (.05), and nursing professionalism (.60), supporting the predictive value of the model. Finally, the model’s goodness of fit, considered an index of general adequacy, both in measurement and structural aspects, was assessed. The goodness of fit value of the current model was .42, indicating a large effect size according to Wetzels et al. (2009) [27].

**Table 4:** Effect sizes of the structural model paths.

LV^a^ impact on dependent variable	R^2^^b^	Impact^c^
Face-to-face interview on nursing professionalism	0.03	Low to moderate
Clinical competence on nursing professionalism	0.04	Low to moderate
Peer review on nursing professionalism	0.40	Strong

^a^LV = latent variable

^b^Change in R^2^

^c^Values of 0.02, 0.15, and 0.35 considered low, moderate, and strong, respectively

## Discussion

Considering the personnel ratio of nurses in medical institutions, along with nursing outcome quality, the development of an evaluation system for promoting competent nurses in an objective and fair manner is essential. Therefore, this paper developed a model for evaluating nursing professionalism and verified the validity of the proposed model.

The nursing professionalism factor that we examined in this study is recognized as a crucial element in nursing. To improve nursing professionalism, it is critical to devise a detailed assessment of both the entirety of the profession and the individual behaviors that comprise professionalism [28]. However, in reality, it is difficult to measure nursing professionalism. In this study, a clinical competence scale was developed to evaluate nursing professionalism. A professional model for evaluating nursing was presented using the dual evaluation systems of face-to-face interview and peer review.

In the empirical model tested here, almost all measures assessing both the indicators and the constructs showed satisfactory levels of reliability, internal consistency, and divergent and convergent validity, supporting the measurement model adequacy. However, in the intercorrelations of latent variables, correlations between the aesthetic and technical dimensions and between the aesthetic and ethical dimensions were slightly higher compared to between the technical and ethical dimensions in the corresponding row. The higher correlations may be explained in terms of the confusing characteristics of nursing practice as an aesthetic dimension or as highly technical education; additionally, fusing the characteristic of nursing as a practical moral action causes confusion [29]. Therefore, the development of an evaluation manual with detailed criteria and standards for all dimensions per item should precede use of the clinical competence scale developed in this study. Additionally, training using the evaluation manual is suggested to confirm a consistent and accurate concept of dimensional evaluation.

The resulting structural model explained 66.2% of the variance of nursing professionalism in the population under study. The model showed great adequacy and predictive power overall and for each path. Therefore, the model accurately reflected the existing relationship between the constructs and their contributions to nursing professionalism in this population.

First, the β path coefficients of the formative measurement model, such as clinical competence and the scientific, technical, ethical, aesthetic, and existential dimensions, presented statistically significant positive correlations; all five dimensions were confirmed as latent variables that configure clinical competence. For the configuration of the clinical competence scale items in this study, content of the focus group interviews was categorized based on the five dimensions of nursing practice [13, 14] and verified through content validation and the repeated indicator approach method. Consequently, the scale consists of items evaluating the following questions: “is proper care that is empirically supported being provided through the use of critical thinking?” in the scientific dimension; “is effective and efficient care being provided that leads to the optimal result?” in the technical dimension; “is ethical and moral care being provided?” in the ethical dimension; “is creative care at the highest level being provided?” in the aesthetic dimension; and “does the care reflect the philosophy of nursing in a practical manner and is it being provided?” in the existential dimension.

The results of the study indicated that the β path coefficient of second-order clinical competence was highest at 0.316 in the scientific dimension, but showed a slightly weak relationship of 0.141 with the existential dimension. However, positive correlations, negative correlations, or indicators with no correlation can also be included in the formative measurement model [30]. This study is significant in that it constructed formative indicators and a hierarchical component model to explain the complex phenomenon of clinical competence and attempted to conduct an empirical analysis. The analysis method used in this study will be applicable for developing tools related to nursing in the future.

Prior studies related to clinical ladders [31, 32] only suggested nurses’ competency or the degree of job performance as evaluation tools for measurement; there was no discussion of multidimensional assessment systems. To address these issues, a clinical competence scale was developed with multisource assessments of peer review and face-to-face interview, and the applied model was suggested for nursing professionalism evaluation. Consequently, the explanatory power of peer review was shown to be the strongest among the three evaluation methods of clinical competence, face-to-face interviews, and peer reviews. Evaluation by peers was the most accurate indicator of success for the individual receiving the review; there were many cases when reviews were used for developmental purposes. Since the review was conducted by a minimum of four to seven peers, reliability of the review increased and had the benefit of removing errors by biased individuals [33]. Nevertheless, the presence of a situational context that blocks honest expression of a peer’s differential performance can lower evaluation accuracy. Unfortunately, there is a lack of peer assessment studies identifying the situational context within a group and the situation surrounding this topic is not yet understood clearly [34]. Such contextual errors were removed in this study, which enabled a strong explanatory power for nursing professionalism with the use of the average score from the peer review process. However, such problems cannot be eliminated, since they may be related to peer review scores used for the analysis of this study. In the case of the peer review, it was only conducted for promotion applicants with more than 70 points on the existing evaluation tool using measurement variables of nursing professionalism. Other missing values were substituted randomly using statistical methods; however, as it may be a result of such methods, careful attention needs to be paid to this interpretation.

Comprehensively, this study is significant as it was the first study to use a nursing professionalism evaluation system for a second-order hierarchical latent variable model in PLS-SEM as a part of CLS development. The evaluation model verified in this study can be used for conducting a fair and objective competency assessment of nurses in the actual clinical field. Furthermore, the rewards that follow the competency of the clinical ladder based on a fair competency assessment can be applied in a positive manner to motivate nurses.

Nevertheless, the following is suggested regarding study limitations. First, scores from an existing evaluation tool were used as indicators of nursing professionalism; however, reexamination of the validity of the proposed model is required after resetting a nursing professionalism gold standard in the future. Second, the score measured in the face-to-face interviews addressed test content validity, and it was scored according to specific criteria. However, interviews have a subjective nature and we cannot rule out the possibility of them being measured differently. Additionally, as the evaluation model proposed in this study only targeted nurses from a single hospital as participants, efforts are required to increase the objectivity and validity of the evaluation system by continuously reflecting feedback from the reviewers and the applicants, as well as reflecting hospital characteristics.

## Conclusion

Nurses account for the largest ratio of personnel in the human resources field of a medical institution. Therefore, it is necessary to secure competent nurses and continuously develop their potential by presenting rewards by different degrees of nursing professionalism. Accordingly, this study developed a fair and objective model for evaluating nursing professionalism, verified the validity of the model, and examined hierarchical latent variables models using PLS-SEM. The reliabilities and convergent and discriminant validities regarding individual evaluations of the clinical competence scale, peer review, and face-to-face interview were consequently verified; the validity of the evaluation model regarding overall nursing professionalism was confirmed as well. Therefore, the proposed evaluation system can be used to evaluate nurses’ professionalism in actual medical institutions from a nursing practice perspective. Furthermore, by providing a conceptual framework of a human resources management system for nurses to manage nursing personnel, the proposed system can contribute to establishing a knowledge system for nursing research and management.
